# Coziness in Games: Second Homes, Audiences, and Esthetics

**DOI:** 10.1177/15554120241310920

**Published:** 2025-01-09

**Authors:** Tanya Krzywinska, Douglas Brown, Minhua Ma, Anton Belinskiy, Kam Bhui

**Affiliations:** 1 Falmouth University, Games Academy, Falmouth, UK; 2 University of Surrey, Guildford, UK; 3 6396 Department of Psychiatry, Oxford University, Oxford, UK

**Keywords:** coziness in games, textual analysis, cozy games, text–player relationship, game culture

## Abstract

The “Cozy” game has gained traction as a concept in contemporary game culture as a set of formal generic features. While we examine the way that Cozy Games are often now defined as a genre, we demonstrate that “coziness in games” has widespread formal and engagement functions and can be experienced in a whole range of games, not simply those defined by the Cozy genre. In focusing on the player–text relationship, and in using a literary model, we seek to widen the discourse and range of meanings of coziness in, as well as of, games. This approach enables us to think about our own lived experience of playing games in terms of the generation of affect, as well as opening the way for renewed analysis of representation, ideology, and cognition.

What does mean to feel at home in a game? Is coziness formal or contextual? Is it a game genre, or can coziness be experienced across a diverse gamut of games? What are its structural and functional aspects? In asking such questions this article seeks to open up a broader, more holistic understanding of the functions and meanings of coziness in games in and beyond the “cozy” game genre. We approach our topic using a literary-driven model focused on the textual relationship between player and game, where functionality and design operate textually. This approach enables us to think about our own lived experience of playing and designing games, providing an experiential understanding of affective grains of playing; the evocative language we choose to use in the paper is very often pitched to communicate something of this. That is not to say that all players experience games in a totalized way, far from it; our article has experiential diversity and situationalism at its heart. As with all relationships, that of the player–text is complex and slippery, since as it is also contextual it opens the way for an additional analysis of representation, ideology, and cognition.

The player–text approach to videogames and their players has its roots in the Humanities, in particular literary studies and film studies. It is informed by reader reception theory (Fish, 1980), formalist analysis (Shklovsky, 1990), phenomenology (Iser, 1972), critical and creative engagement with texts, and semiotics (Barthes, 1975, 2000, 2004). While social science methods and empirical player studies offer valuable insight, our text–player approach looks to the experience of playing games through relationships with the game text and its formal strategies. While coming at the player–text relationship from different angles, various game studies’ authors have helped to establish the text–player approach. Aarseth (1997) called for a formal approach to games, while Atkins (2003) brought literary and reception theory to advocate the role of the player as reader. Carr (2006), Krzywinska (2006, 2015), Mäyrä (2008), Vella (2015), Fernández-Vara (2019), and Meakin (2023) have each argued that a player–text approach to games enables a formal *and *experiential analysis of individual games that helps understand how they create meaning and affect. As Atkins and Krzywinska (2007) propose, game studies should be pluralist, particularly given the disciplinary breadth videogames cross and therefore tolerant of diverse approaches and methods. In this article, our decision to focus on the player–text relationship is based on a need to be highly sensitive to context and difference meeting with shifting meanings in terms of interpretation, ideology, and representation. This allows for a highly situational understanding of the functions, meanings and experiences of coziness. It is within the complex and unstable nature of player–text relationships that “coziness,” in its various forms and locations, is found. For these reasons, we draw from a range of sources (videogame, player, diegetic, and paratextual texts) to help build our argument around a polysemic, situational, and critical conception of coziness.

As a genre, Cozy Games grew out of a player-led collective perception that certain games felt more “cozy,” “zen,” and comfortable to play than others. To some extent, this created a distinction that marked certain texts and play experiences off from intensively combative games. Requiring speedy reactions and keen alertness in response to rapid real-time changes and cues in the visual and auditory field, such games do not give players time to plan or linger. Working with this established “hard-core” modality, some games market their difficulty as a positive feature to a well-defined and understood gamer market. As games have become more diverse, they have reached new and potentially lucrative audiences. Games designed to educate, train, or raise awareness are also increasingly prominent in the busy gaming marketplace. In this context, a category of games has emerged that actively employ formal features designed to provide players with welcoming and comforting experiences. Cozy Games are not synonymous with “casual games,” however, as they demand substantial amounts of time and knowledge from their players. Compared to games like the “Dark Souls” franchise, self- or community-genred Cozy Games can be characterized by their relaxing, forgiving, and nonthreatening gameplay, a particular aesthetic approach, and their emphasis on community-building (Cook, 2018; Waszkiewicz & Bakun, 2020). As we argue, this recent nomenclature does not however give Cozy Games sole ownership of “cozy,” and there is more continuity with some staple game genres than might otherwise appear. As we will show, the roots of Cozy Games are clearly apparent in other genres, and often a cozy aesthetic is used to create for players at certain junctures and designated spaces in a game to provide players with optional respite from the hurly-burly of intensive gameplay. What the article seeks to demonstrate therefore is that coziness has a role to play in games generally. This is grounded in the text–player relationship: it can be formal—ie a property of a game's overall design, but it can also be located more discretely within the experiential, something that comes out of an individual player's gaming experiences and personal history. While we do spend time mapping out the formal coordinates of coziness in games to show how it can work structurally, we will later move on to focusin more detail on coziness as located within the player's lived experience of playing games. We do however want to outline key aspects of textual and gameplay vocabularies that have become associated with coziness before going on to argue for a broader understanding of coziness in games, an aspect that has so far been sidelined by academic literature around Cozy Games.

## Definitions of Cozy

To usefully come toward the values of “cozy” it is best to start beyond the boundaries of games, with the conceptual. Concepts such as “Hygge,” “Heimlich,” and “Cozy” are all connected around a sense of “homeliness,” indicating belonging and comfort. In their different guises, they relate to the way space is categorized along cultural and psychological lines. Mary Douglas’ seminal anthropological analysis on the cultural division of space, *Purity and Danger* (1996), points us to the way that “home” has a structural role to play in how space is designated and meaning assigned. The special psychological status of home must be protected, and it further defines and decenters “not home,” i.e., that which is other to home. Literature on the experience of the “Uncanny” (Freud's “The Uncanny” 1919 [1989]; developed by Fisher, 2017) indicates that *homeliness* is easily made uncomfortable by becoming wrong, out-of-joint, disrupted, spoiled or, as Fisher puts it, when there is an absence of presence, or a presence of absence. In the psychological literature, *home* has a fundamental role to play in defining civilization, identity, and centrality: what is felt as *home* is all at once of psychology, culture, social, as well as semiotic and structural. While psychological grounding is inherently linked to comfort, stability, identity and family, the cozy status of home is fragile. It is something to nurture, reinforce, and protect; it is not “home” in a passive way but must be vigilantly reinforced as a place to live and feel secure. This is why the concept of home is so potent ideologically and implicated in exclusive and inclusive rhetorics of Otherness. Many different fictions make use of the concept of home to serve narrative and dramatic purposes. Indeed, any contrast with home and its coziness has the capacity to be narratively potent, creating an anticipation of adversity or adventure. In this sense, coziness has an important dramatic structural function. Tolkien's *Lord of the Rings* trilogy provides a useful example.

The trilogy opens with the routine daily life of hobbits, revolving around hearth and home, the growth and consumption of *hearty* food and drink. Theirs is a supremely cozy life: hobbit homes are round, perfectly formed for their lifestyle, and full of homely comforts. This idealized, rural, and very human conception of the cozy home is the precious thing that is fought for against the cold dehumanizing might of Sauron. “Bag End,” that coziest of hobbit homes, represents all that is good; even though it must be absented to preserve it from becoming “spoiled” by “filthy” orcs (Tolkien's ‘othering’ language reflecting Douglas’ purity and danger model). The potential pollution of the cozy hobbit home (a bucolic and normative ideal) is what motivates Samwise Gamgee not to give in when all seems lost and hopeless. In terms of dramatic structure, the cozy home and the life it represents lend shape and purpose to the story and character arcs. The centralized placement of the cozy home is a staple of games, signified semiotically and spatially, proffering the thesis to the antithesis of conflict and unrest. Cozy Games seek such centrality.

## Defining Coziness in Games

In broad terms, we want to distinguish those games that are generically identified as *Cozy Games* from those games drawn from across genres that use cozy elements as structural components. In addition, we will go on to show later that to broaden the definition of coziness in relation to games we need to look at what is comforting, homely, and cozy in terms of the lived experience and associated affect of playing games. As such, we advocate the position that “cozy” is not simply a genre, but instead has a profound presence in the player–text relationship. In focusing on coziness in and of games, we turn our definitions toward the complex problems of self-care in the digital era. Understanding the psychological and social currency of cozy in games can potentially offer insights into the role that games might play in contemporary culture as havens from (unhomely) homes that might be unsafe or frightening.

The nomenclature “Cozy Games” has been used quite widely in relation to Nintendo Switch games (although these games are often multiplatform). The Youtuber Eeowna is an influencer who makes consistent use of the term “Cozy Games” as a means of reviewing a somewhat diverse group of games and has consolidated this over the course of several broadcasts (https://www.youtube.com/watch?v = 8g3lOMtFNmQ, accessed 15/08/23). Eeowna's definition provides a useful position from which to grasp something of what is commonly referred to as Cozy Games. It should be noted that the configuration of genre is quite complex in the context of games, referring to a range of different properties: they may draw on those established in other media (SciFi, Western, etc.) or describe gameplay type. In tune with this observation, Eeowna's use of the term *Cozy Games* has multiple components. First, she locates “cozy” in terms of her own experience and focuses on games that don’t demand intensive gameplay. She also acknowledges that coziness is subjective, pointing out that an example of what she regards as a Cozy Game, *Dave the Diver* (Mintrocket, 2023) (Dave is a fish-spearing diver and sushi chef), may not be seen as such by players who find the fish hunting element of the game ethically problematic, creating for them an “uncozy” gaming experience.

Second, she identifies two gameplay modalities that are core to her definition of the genre: Farm Sims and RPGs (role-playing games). Visually Eeowna's conception of Cozy Games follows established patterns of “cute” (typically animals with big eyes that look like huggable soft toys with rounded shapes) and the use of relaxing ambient or folk music. Most of the games she claims as cozy are also made by or in a Japanese style and reference Japanese culture. While a few of the games she references have dark themes, it is *friendship* and *community* that are centralized—taking their cue from Farmville-type mechanics where friends (in-game or other players) help with the task of farming (https://www.youtube.com/watch?v = 8g3lOMtFNmQ, accessed 15/08/23).

Eeowna identifies two games as the source for the burgeoning development of Cozy Games: *Stardew Valley* (2016) and *Animal Crossing: New Horizons* (2020) [abbrev. to SV and AC:] and which consolidate cozy textual patterns. AC is also a game that Clüver et al. (2022) argue offers players self-care activities and means to escape the stresses and complexities of modern life, seen as a core component of Cozy Games and promoted by formal textual features.

For Clüver et al. (2022) AC's nonlinear gameplay, lack of competitive pressure, and emphasis on player personalization are key to its function as a self-care tool. Activities such as fishing, gardening, and interacting with anthropomorphic animal villagers encourage mindfulness and relaxation, as well as creating a sense of community and belonging. Furthermore, the authors highlight the importance of social interaction, demonstrating how the game's online multiplayer mode enables players to maintain social connections and engage in acts of kindness and cooperation. They argue that these interactions promote a sense of well-being and help alleviate feelings of isolation; as other authors have noted, this was particularly the case for such games during periods of social distancing and quarantine in the COVID pandemic (Barr & Copeland-Stewart, 2021), and (Lewis et al., 2021). Fernandez & Moreno (2023) go on to argue that *AC:NH* is an especially inclusive space.

A set of design principles that has been influential in the definition of Cozy games is a group report (Cook, 2018) authored by a think tank of professional game designers which outlines a set of design pillars for Cozy Games:

“**Safety**: A cozy game has an *absence* of danger and risk. In a cozy game, nothing is high-risk, and there is no impending *loss* or *threat*. Familiarity, reliability, and one's ability to be vulnerable and expressive without negative ramification all augment the feeling of safety. To maximize safety, activities should be voluntary and opt-in so that players never feel the threat of coercion. **Abundance**: A cozy game has a sense of *abundance*. Lower level Maslow needs (food, shelter) are met or being met, providing space to work on higher needs (deeper relationships, appreciation of beauty, self actualization, nurturing, belonging). Nothing is lacking, pressing or imminent. **Softness**: Cozy games use strong aesthetic *signals* that tell players they are in a *low stress* environment full of abundance and safety” (https://www.projecthorseshoe.com/reports/featured/ph17r3.htm, accessed 12/07/24).

This Cozy Games manifesto raises some interesting challenges to the way that games have themselves been defined. Designer-academics such as Salen & Zimmerman (2004) and other more form/structure-focused game academics (Bogost [2006], Galloway [2006]) have worked with the idea that games have rules that structure action and are therefore procedural in nature. The types and functions of rules differ of course, with some are very much more exacting than others depending on context. Philosopher Bernard Suits argues that games are full of “unnecessary obstacles” which are the constitutive components of any game: we accept rules because they make gameplay and a sense of challenge possible (1978, 34, 41). Roger Caillois argues that it is the rules of games that differentiate them from freeform play (2001, 9–10). Cook et al.'s blueprint moves us away from the “hardcore” end of the game spectrum and toward the “freeform” play end of the spectrum, yetthere are still rules and proscribed activities in Cozy Games, as listed by Eeowna. As such, Cozy Games remain within the “magic circle” of games but without moving off into the sphere of triviality or the type of freeform play offered by sandbox “games.” What Cook calls for is avoiding mechanics (rules) and stories that lean toward strife, intensity, and competition. By contrast, our argument is that coziness is not so strictly defined and limited to a genre; it is found in many different games where it has structural and dynamic functions as well as created through players’ relationships with the games they play. As such coziness may well be experienced when playing games in which competition and intensity are strongly present. .

The guiding principle that emerges from Eeowna, Cook, and other Cozy Game scholars is that a core function of a Cozy Game (or indeed space) is as a refuge from a complex and pressured world, thereby promoting self-care through low-key gameplay mechanics and a gentle, nurturing atmosphere. Resource management is present in most games that Eeowna claims as Cozy Games, but what makes this cozy is that it is carried out without any sense of competition with other players. In both Eeowna and Cook's definition, it is how resources are contextualized in terms of gameplay and story space that divides the cozy from the non-cozy.

Two main questions arise from this however: does a corralled definition of “coziness” in game design miss out on the way other games create engaging rhythmic and differential patterns of gameplay where coziness plays a role? And, do Cozy Games’ anodyne and idealistic evocations of homeliness fall short in comparison with more dynamic and affecting game-based storytelling? Losing the “game” from games might be just another road *en route* to a culture that is constantly in avoidance of the complex and varied nature of experience. Before we can begin to take on such issues, it is important to establish the presence of coziness across the breadth of games to move usefully beyond the focus on Cozy Games as a proscribed genre.

## Cozy Game Aesthetics and Mechanics

So far in this article, we have described family resemblances across games that can be classified as Cozy Games. We have also isolated two key games which characterize the patterns which can be said to make up Cosy: *Stardew Valley* and *AC:NY*. Establishing the main coordinates of these patterns across gameplay and audio-visual aesthetics in these and other “Cozy” games will also help with showing up how coziness manifests in games that don’t present as Cozy Games. This examination is in the wider service of our overall argument that “coziness” is not limited to a genre. This section of the article explores the components of cozy beginning by breaking down the genre signifiers by focusing on mechanics and aesthetics, moving on to customization and story, and finally the key concept of the “home space.”

First off, the Cozy Games mentioned thus far are available on the least expensive platforms. Although available in more traditional PC and console setups, they can be played on mobile platforms. *SV* is now available on portable devices, as a mobile phone app and on the Nintendo Switch console. As such the games can be played sitting on a sofa in a position of ease and conferring a particular felt state. Cozy Games could be legitimately regarded as “Sofa Games”: you don’t need to sit bolt-upright at an expensive rig to play them. Accessibility and aspects of the casual are then principles that underlie Cozy Game textual and design strategies, lending them a wide potential market. Cozy Games have what we might think of a *low-key* gameplay design. That is not to say they aren’t involving for players or that they don’t have depth, however, while simultaneously decentralizing activities involving conflict or intensive active planning. Indicative of such a ludic aesthetic is the “pet battle” component of *Ooblets* (2020) where battles are symbolized as dance. SV focuses on farming and low-key resource management, and includes, but does not foreground, a combat system in which defeat has no serious ramifications or fail state. In addition, nothing is time critical or demands group play to access content. The emphasis is on exploring, collecting, and making friends, with no win state or one-time missed opportunities.

In tune with the ambiance of a relaxing game, the semiotic formations of these games’ mise-en-scene are underlined by their employment of ambient music and sounds, often using organic sounds and acoustic sounding instrumentation. Visually, it is earth colors that provide the signature of the color palette: in the case of SV, yellow ochre, and warm browns. In terms of colour theory, warm colours abound, even the greens are mainly on the yellow, and therefore warm, side of green. Pops of orange-red are also warm, with only the pale pinks and the odd fir tree working on the cool blue spectrum. Warm colours are cozy in the signifying schema of SV and these are also highly saturated, vibrating richly from the screen as a form of optical welcome. Even though AC has a wider colour palette and employs 3D, it is still warm colours that prevail, blended into a diffused optical softness. The more recent *Cozy Grove* (2021) has a distinctive “indie” visual style. Here colour is instrumental to gameplay and operates as an important and impactful feedback mechanism, working strategically with warm and cool colours, as well as employing desaturated earth tones. As the player collects the logs needed to feed their friend and guide, named Flamey (warmth and comfort embodied in this cozy fire character), bleached-out cool whites and pale blues turn more colourful becoming carefully toned tertiaries as if painted with graduated cloud-like watercolor. This desaturated environment is calm and gentle creating signifiers of coziness through the flowing hand-drawn inking. Pastel colours, earth colors, hand-drawn imagery, or 8-bit game esthetic nostalgia are all leveraged in their gameplay contexts to construct a normative visual language of coziness.

Added to this is the use of cute imagery and magic. While many Cozy Games employ farming simulation mechanics, the realism of the simulation is not foregrounded (as with *Farm Simulator* [2008–2023], for example); instead, they are coded as fantasy spaces. That does not suggest that realist simulations can’t be cozy for players however but here we are focused on games often made within the terms of Cook et al.'s Cozy Games manifesto. Characters with large eyes abound (even achieved in the 8-bit visuals of SV), as do cute animals and magical creatures. Even the vegetables are cute, or fire (!), as with the character Flamey in *Cozy Grove*. As outlined in the Cook et al.'s manifesto, minimizing threat is a design pillar of the genre. It is not true to say that sadness and loss are debarred from Cozy Games, however. SV itself deals in well-concealed themes of war and addiction when you get below the surface of several characters (Op de Beke, 2021; Mackay & Roberts, 2023), while *Cozy Grove, Beacon Pines* (2022), *Sky Children of the Light* (2019) and *Spiritfarer* (2020) each focus on moving-on the spirits of the dead, taking their cue from Studio Ghibli's film *Spirited Away* (2003) and Japanese folklore. Magic is often leveraged toward the construction of the fantasy space, as in *FaeFarm* (2023), and is designed around its benign use and often associated with rural culture and the pastoral–closeness to nature often playing its part in the Cozy Games recipe. In the context of the genre, magic is often used to create gameplay scenarios such as making imaginary friends, creating enchantment through visual effects, providing the basis for gift exchange, thereby creating a particular form of escapist agency in the game-world.

## Constructing Coziness in Games Outside the Cozy Game Circle: Hogwarts Legacy

As we’ve now established, coziness in Cozy Games is defined around the generation for the player of a sense of comfort and belonging within the game environment (Fernandez and Moreno, 2023). We’ll now look away from the Cozy Game genre toward formal structural uses of coziness in games that are not designed to be Cozy Games. *Hogwarts Legacy* (HL) (2023) is an open-world game available across multiple platforms based on the Harry Potter franchise that draws on Role Playing Game formats. What drew our interest to the game for this article is the extensive player customization options that enable players to create a character that reflects their own identity or aspirations. This includes choosing their character's appearance, magical abilities, and house affiliation. We put forward the idea that textual customization could be one way that players become more at home in-game, enhancing a sense of belonging and coziness in a game space. We are moving here toward a notion that such certain mechanics used in games that are not defined as Cozy Games can provide an experience of coziness. As Behr et al. (2015) and Isbister (2016) among others, have argued, *customization* allows players to become more immersed in the game world and feel a greater sense of connection with their character's journey. In a (real) world, where there is often little choice and agency and where a sense of being “at home” might be something distanced and remote, the “cozy” appeal of choosing your own special powers and appearance has some obvious attractions and which may well act in compensation for, for example, feelings of insecurity and detachment. The ability to create a character or living space within a game that represents a safe and empowering identity (Klimmt et al., 2009) may help players feel more like an active agent within the game rather than having to fit into an un-cozy predetermined character which might not feel representative. As well as the cozy potential of customization in HL, a sense of coziness might also be experienced through the nurturing and supportive atmosphere of the Hogwarts school environment itself with its various mentors, friends, and magical creatures, perhaps providing an additional layer of comfort for players seeking refuge from real-life challenges and seeking positive messages of personal growth and development. Facilitated by player customization, the growth storyline plays a key role in the game's construction of a framing milieu of coziness. More than this, HL's componentry of coziness facilitates engagement with darker themes while also pointing toward resilience, empowerment, and agency.

The Harry Potter (HP) novels and films engender for many readers (and particularly young readers) a warm sense of belonging. The game leverages that “cozy” textual and experiential capital as a buffer which, as with the books, enables in a structural way the dramatic narrative focus on, for example, death, morality, and struggles relating to power, family, and identity. As an adaptation of a very popular and widely known franchise, *HL* draws on familiar and well-established iconography and visual motifs, story arcs, and characters. The game does not attempt to divert from those patterns, as Stobbart argues of franchise additions such as *Bioshock* (2018, 386). For a generation of gamers raised on Harry Potter, these games evoke an equally powerful nostalgia, riffing off of connected aspects of homeliness and familiarity. The player's history with the HP franchise connects their lived experience with the textual strategies used to create a sense of coziness. The game's model of customization is designed to be in keeping with the franchise, freedoms are therefore limited, and it is a tool used to locate the player in the textually proscribed gamespace, persevering the franchise's consolidated definition of cozy. It is also worth noting that even where customization is far more open, it might not aid in the process of creating a sense of coziness for players. Proffering freeform customization creates a barrier to, rather than an invitation into, a cozy space (Hunicke, 2005).

Highly customizable sandboxes and digital dolls’ houses rely on the curiosity of a player to find things out rather than being presented with a set of clearly delineated tasks and winning conditions. However, in both cases the ludic proposition is into a game's textuality—we “read” it because it is semiotically loaded; it is how game space creates meaning. We propose that the predictability and reliability of a game's ludic structure are intrinsic to the experience of coziness, regardless of diverse themes, story and iconography. For example, in games played many times and with proficiency, the ability to be “in-control” in a familiar “known” space makes for a pleasing experience of purpose and ease. We now turn to this aspect of the text–player relationship in a discussion of the powerful connections between familiarity and coziness as it is an area that tends to be elided when the focus falls on Cozy Games as a genre.

## Games as Cozy Spaces: The Home Space

While Cozy games and games such as HL carefully design their textual modalities as “cozy,” they do not have monopoly on coziness. We propose that the experience of coziness, of feeling “at home,” in a game has something to do with the very nature of game worlds. To unpack this notion: games are ludic textual spaces that are fully oriented around the player or virtually co-located groups of players and their sphere of actionable agency (Krzywinska notes the Vitruvian qualities of games, 2015). For example, terrain is designed around player actions, semiotic signposting throughout the game marks player pathways and points of interest aiding in the navigation of game space and what is to be done there. In addition, arrays of feedback data marking a player's progress are built into the textual fabric of the ludic space and the user interface. The requirements needed to “win” at either small or large scale are often spelled out both semiotically as well as built into a game's physical controls (Galloway's notion that all games are codified and that games are actions, with diegetic and nondiegetic controls, 2006). From our text–player perspective, games are by nature knowable, textually motivated spaces. It is this that makes games cozy for some players—players who have learned the ropes and feel included by these aspects of games which address their literacies and consumer capital, even where a game sets out thematically to excite, subvert, and disturb (the Dark Souls franchise provides a good example). We need then to look in more detail at the twin principles in play *predictability* and *familiarity* if we are to build a broader sense of coziness in and of games.

The systemic predictability of, and player familiarity with, what the ludic textual space requires of the player is intrinsically linked to an experience of coziness—a game is defined around the demand for certain sets of often well-rehearsed actions, which then lead to known consequences alongside hard-wired ritualized actions (with some variety thrown in to keep the player actively engaged). This coziness is sourced from within a player's experience of games; it is connected to a flow state, but founded in a sense of knowing where things are and what to with those things to produce a desired effect, as defined by the game's hard-coded procedurality. This experience is comparable to the ways we create and manage the coziness of our own homes—i.e., knowing where things are without thinking about them, knowing the way to the bathroom in the dark, where the light switches are, etc. Both provide in their way bulwarks against the unpredictable and the strange (plague, war, illness, injustice, inequality, entropy, and attrition).

We can see this player–text relationship modality at work in horror games. While horror games ostensibly seek to disrupt homeliness in a thematic sense, they often rely on well-worn conventions of gaming that prescribe for the player when and how to act to manage diegetic threats. Horror games can therefore feel like home to a player who knows and enjoys the patternings of control and chaos they offer (Perron, 2018). Conversely, where games feel to players that they lack security in terms of a game's design integrity or through a lack of good player onboarding, making a game feel unpredictable and/or unfamiliar, then they can feel deeply uncozy. Horror titles masquerading as more casual genre games such as *Inscryption* (2021), which mimics a card-battler, or *Doki Doki Literature Club* (2017), ostensibly a visual novel, are testament to the power of this effect. Games of whatever stripe optimally provide orchestrated and secure spaces made to support a player to navigate and progress through the game, even where that is tested, to create drama and a sense of achievement.

Coziness in games is therefore established in multiple ways: from user interface design and gameplay to the use of feedback mechanisms, spatial design, and audio-visual conventions. *Coziness is thus more than a genre*: it is also sourced within the nature of the videogames and in the player–text relationship. And, as with HL, many diverse games outside of Cozy Games create formal features to instigate that experience.

As stated, the cozy home of hobbits plays a key structural role in Tolkien's *The Lord of the Rings,* a body of work that shaped role-playing game conventions (Peterson, 2012). Many real-time persistent world-based games follow suit and tie 'home' into their spatial construction. The MMO *Everquest* (1999–) granted players a personal house, a base; while *World of Warcraft* had no direct equivalent, in the “Warlords of Draenor” expansion, players had a garrison home that could be customized and within which NPCs could be interacted with and acknowledged players’ diegetic achievements, creating a sense of community, marking and centralizing the player's history within the long form of the game. Other players could be invited into these personal spaces offering spaces of respite for socializing, crafting, and farming resources. *LOTRO* took another approach to “home space,” perhaps taking a cue from *Second Life*'s real estate model. Individual kinship homesteads can be rented, with a choice of styles and sizes. Each has an address and rent paid on it. Players can decorate the space with crafted materials, or mementos acquired in adventures and fights. Borrowing from the audio found in the game, music choice is also an inbuilt feature, creating atmosphere and every house has a cozy hearth and flickering fire. These textual, semiotically loaded spaces support a sense of belonging in the world (locating the player as *of* the world) and encourage players to return to these spaces as a base as well as supporting role-play and group activities outside of core gameplay. They are prescribed spaces: what is done and placed within them are dictated by the procedural nature of the game—coziness therefore being defined by the semiotic and ludic logic of the game's world. Many games for example have other rest areas, such as the cozy inns of *WoW* or the fireside camps for enforced periods of rest in *Baldur's Gate 3* (2023).

For many fans, the worlds created within *WoW* or *LOTRO* provide players with their fantasy second home: a normative and prescribed “safe” place to dream, play, and be creative in a guise and role that is determined within the parameters of the game. Here *cozy* is tailored and constructed through the player's *imaginative engagement* with a game's supportive ludic textual milieu, and this can go beyond limits usually proscribed. LOTRO's kinship houses for example are large stage sets designed to support groups in their role-play, which might run counter to the set text. Such affordances could be said to make this version of cozy more of an open *possibility space* than that found in the more fully realized, pre-ordained version of “cozy” that is found in Cozy Games. Texts can be read in many ways and role-play can run counter to the set text, yet it is always the case that actions in games are prescribed and procedural even where players find ways to play with and against the text. Many open-world games also support player communities and the presence of others in a game or on related fora can potentially create a sense of game-based coziness even as many have experienced the exact opposite.

### Cozy Communities

The section above refined the broad experience of coziness into a much wider array of aesthetic and mechanical features, finding it in the predictable form of games more generally. Some games create diegetic communities for players, which are highly controlled, creating their own ecosystems for coziness. Player-based communities construct fertile yet more unpredictable grounds for the generation of a sense of belonging and coziness. There are many fan and player studies within game studies, some based on ethnography (for example, Taylor (2006); Williams et al. (2006: 342). Ours is not a player study in the sociological sense; however, our lens on coziness in games is through the complexities of the player–text relationship. Community in its most positive guise can create a sense of belonging and inclusion based on shared co-located experiences and a common set of references (Saldanha et al., 2023). Game culture is however far from a comfortable place for many, with its tendency towards exclusions and exclusionary behaviours. These can take the form of issues of accessibility (Carr, 2014; Anderson, 2024), or of representation, otherness, and difference (Mukherjee, 2018; Buhari, 2024; Iantorno & Consalvo 2023; Kampmann Walther & Larsen 2022). Exclusion is practised through trash talk, a heightened senses of competition and self-aggrandizement, displays of mastery, and disputes. Bullying and belittling speech are rife, aided by anonymity and “game” capital. Many players, these authors included, have however experienced supportive, thoughtful, and community-oriented forms of behavior, albeit most likely in localized group contexts (ie guilds). Many multiplayer games have a range of measures in place to limit such behaviors, with terms of play set out in User End License Agreements. These include a range of in-game management techniques and peer-based methods. While the “toxic” nature of game culture rightly receives a lot of press, there are then other stories to tell about community support, friendships, and other positive experiences (Williams et al. 2006).

In games made specifically to be cozy, nonplayer characters are designed to be supportive and give the player a sense of having in-game friends. Many of these games represent (ideal) communities too—reciprocity being the major keynote; creating a safe and controlled simulation of a community and friendship network particularly where the community is entirely textual. Where games are online and offer the ability to play with friends, the gameplay is often focused around cooperative play, working together to exchange goods or work together to gather or collect resources (as in *AC*, *Farmville*, *SV*, etc.).

Multiplayer Online Games (MMOs) by contrast create their communities through the social nature of the game space, through gameplay types, social communication channels, and shared events–while these facilitate a “virtual” community, they are also *of text* in some way or other, making for a different textually mediated modality. In many MMOs, some content (loot, story, and achievements) is only accessible to those playing in groups which contribute to the formation of player-made groups: Clans, Kinships, or Guilds (as in *Final Fantasy*, *WoW*, *LOTRO*, etc.) each place emphasis on community built around reciprocity, care, and shared experiences, and supported by the ludic textual features of the games (i.e. Williams et al., 2006). Some *WoW* guilds have been going for many years and provide social and emotional support facilitated by both their longevity and the shared experience of playing together for mutual benefit (Chen, 2009). Guilds often also work to provide player-based policing of behavior, interpersonal sanctions, and ultimately players can be “kicked out” of a guild. Trolling and toxic play are part of game culture, but there are many other methods of socialization in play to create a sense of belonging, community, and safety.

While Cozy Games are often walled off by developers from other games, there are some strong family resemblances. Many Cozy Games utilize MMO and role-playing game elements to create a sense of belonging and community, with AC as a good example. The game leverages real time toward this, enabling players to meet in the same space and limiting the “speeding up” time mechanic in service of this end. In addition, many of these games often refer to real-world calendar-based festivals to create events that tie to real-world time and events (WoW's Hallow's End, Winter Veil, etc.) providing material for role-play as well as shared experience. This alignment with real-time has been a core feature of the many games in the AC series, often making use of console clocks to determine day/night cycles and festivals. The reference to real-world festivals also gives the games a sense of “Once upon THIS - *shared* - time”—co-locating players together in time and place, giving a sense of connection through the shared experience of temporal presence and cross-cultural ritual.

Creating bonds between player characters is also something that Cozy Games borrow from RPGs and MMOs. In the early days of *Everquest* enterprising players would tout their wares to others in the town square–setting up gambling stores, selling potions, dyes, and other items they had gathered or crafted and setting their own prices. Later MMOs often sought to rein in such ad hoc player-led economies, replacing with economic mechanics such as auction houses. In MMOs, player-to-player exchange of items and money is a key to understanding what constructs a sense of community and sharing; of the creation of a “real space” and a shared, mutually beneficial, economy. Coziness therefore originates out of the development of in-game economies even if that is sharing in a gift economy with friends.

All this social economy in support of social coziness is of course facilitated by the various textually mediated modes of communication enabled by the games. Some players use extra-game channels, such as TeamSpeak or Discord. In-game channels are multiple in most MMOs–ranging from direct text whispers to server-based trade or group text channels (again in *WoW* these are limited to faction thus helping to contain faction rivalry to spilling into more personalized rivalry). Creating a sense of community in all these games is therefore embedded very deeply in the player–text relationship; coding deployed to control and encourage a sense of textual-motivated cross-player sharing, exchange, and communication. Games are highly structured, codified, and policed spaces - predictable and masterable , and as such experiences of coziness can be found here as a general proprety of games, even if that offer is contigent on the nature of an invividual player-text relationship.

## Concluding Remarks

In breaking down what seemed an obvious “cozy genre” into component parts with key themes supported by an underlying social dynamic, we’ve shown that “coziness” has a variety of forms and functions with no single formula. The cozy effect is reached therefore in different ways suited to context and audience. Cozy Games do not have sole ownership of this device, and their design vocabularies of coziness are specific to their audiences. In asking “what and whose coziness,” we go some way toward opening it up to difference, demonstrating that coziness has an inherent, if contingent, role in games through the text–player relationship. The experience of coziness is however never guaranteed or incontrovertible in any context: *Hogwarts Legacy* might prove cozy for some but not for others; those who take issue with JK Rowling's comments on trans people the Potter-verse cannot now be a cozy space, when once it perhaps was.

Through this critical investigation we have come to regard coziness as more central and integral to games than we originally suspected was the case. Coziness in and of games has a formal, social, and psychological role and is generated through the player–text relationship. While its status is subject to change, the experience of coziness in games is in part based on procedural predictability and semiotically loaded player-centric spaces. Predictability and familiarity are key componentry: whether that is with game conventions, in gaining interactive access to a well-loved fiction, or in terms of a managed social space. The cognitive framework provided by the status of games (as fiction and as a game) cordons them off from the real world, at least to some extent, creating what Caillois called the magic circle of games and where they can become symbolic, narrative, and transformative spaces; as well as perhaps a chosen second home. All kinds of games offer the cozy potential to leave normal life at the door to engage in imaginative, creative, and proscribed activities. As a form of ludic onboarding, storytelling in games enfolds the player into the game space and its affordances, perhaps creating a safe and cozy context for players to test out new emotional or social situations. In this, we go quite a long way beyond the narrow definition of Cozy Games outlined by Cook's blueprint to posit the design idea that a positive and enriching game experience is best supported by a well-made balance between coziness and darkness, between ease and focus, familiarity and novelty, customization and proscription. Creating through ludic textuality a cogent sense of “being there” while providing distance enough to “play” knowing this is a safe space to do so.

One of the purposes of this article was to aid in our project to develop a game aimed to provide a safe space for young people who have experienced trauma and in so doing allow them a space for both enjoyment and emotional processing. Once that game is completed, writing up the process and its design decisions is the topic for our next scheduled article. What we hope to have shown here however is that coziness resides in the player–text relationship and that it has key psychological as well as formal and structural functions.

We are very aware that an overly anodyne, heavily controlled, and idealized safe virtual space might be regarded as a way of proffering an Orwellian pacification: seductive, bucolic, and infantilizing; setting impossible expectations and disavowing the inevitable googlies of life, as Harrington (2015) argues about Disney films. We are also aware that social economies of taste and cultural capital are very much in play in regard to the different routes to “coziness,” what is cheesy for some, is super cozy for others; identity politics and peer groups play their roles in this of course, particularly in the tribal context of games. This is of course an old debate regarding the purported soporific qualities of popular culture viz the Frankfurt School. Dyer's (2002) take on the musical does however make the cogent point that in times of strife, loss, and discomfort, losing yourself in an entertaining confection can recharge us, conserving or releasing the energy needed to carry on the daily grind. Games provide many cozy modalities and are not limited to the cute, ambient, and analgesic. Games of all kinds are proving to be a method for cushioning against chaos, unpredictability, isolation, inadequacies, and diminished agency. For some, coziness is found in a competitive drive and intense concentration; while others want a relaxing engagement; in games and in both cases progress is guaranteed and the formula for success is clear, and this is instrumental to the player–text creation of cozy in whatever genre.
